# FTO gene polymorphisms and obesity risk: a meta-analysis

**DOI:** 10.1186/1741-7015-9-71

**Published:** 2011-06-08

**Authors:** Sihua Peng, Yimin Zhu, Fangying Xu, Xiaobin Ren, Xiaobo Li, Maode Lai

**Affiliations:** 1 Department of Pathology, Zhejiang University School of Medicine, Hangzhou, P. R. China; 2 Department of Public Health, Zhejiang University School of Medicine, Hangzhou, P. R. China

## Abstract

**Background:**

The pathogenesis of obesity is reportedly related to variations in the fat mass and an obesity-associated gene (*FTO*); however, as the number of reports increases, particularly with respect to varying ethnicities, there is a need to determine more precisely the effect sizes in each ethnic group. In addition, some reports have claimed ethnic-specific associations with alternative SNPs, and to that end there has been a degree of confusion.

**Methods:**

We searched PubMed, MEDLINE, Web of Science, EMBASE, and BIOSIS Preview to identify studies investigating the associations between the five polymorphisms and obesity risk. Individual study odds ratios (OR) and their 95% confidence intervals (CI) were estimated using per-allele comparison. Summary ORs were estimated using a random effects model.

**Results:**

We identified 59 eligible case-control studies in 27 articles, investigating 41,734 obesity cases and 69,837 healthy controls. Significant associations were detected between obesity risk and the five polymorphisms: rs9939609 (OR: 1.31, 95% CI: 1.26 to 1.36), rs1421085 (OR: 1.43, 95% CI: 1.33 to 1.53), rs8050136 (OR: 1.25, 95% CI: 1.13 to 1.38), rs17817449 (OR: 1.54, 95% CI: 1.41 to 1.68), and rs1121980 (OR: 1.34, 95% CI: 1.10 to 1.62). Begg's and Egger's tests provided no evidence of publication bias for the polymorphisms except rs1121980. There is evidence of higher heterogeneity, with *I*^2 ^test values ranging from 38.1% to 84.5%.

**Conclusions:**

This meta-analysis suggests that *FTO *may represent a low-penetrance susceptible gene for obesity risk. Individual studies with large sample size are needed to further evaluate the associations between the polymorphisms and obesity risk in various ethnic populations.

## Background

Obesity is becoming a worldwide epidemic in modern society. It is prevalent in individuals of both genders and of all ages, socio-economic strata, and ethnic groups. It is estimated that the total number of overweight adults has reached more than 1.1 billion worldwide, including 312 million obese individuals and that about 10% of children are classified as overweight or obese [1,2]. The worldwide obesity epidemic is mainly caused by environmental factors, including excessive food intake and a lack of physical activity [3]. Obesity may lead to cardiovascular disease, type 2 diabetes, and several cancers. Overweight and inactivity account for an estimated one-quarter to a one-third of cancers of the breast, colon, endometrium, kidney, and esophagus [2].

In 2007, *FTO *(fat mass and obesity-associated gene) was first discovered in a genome-wide association study (GWAS) for type 2 diabetes [4], and, almost simultaneously, two other teams independently reported that the *FTO *gene was associated with obesity (or obesity-related traits) in a GWAS and a genetic association study [5,6]. The *FTO *gene, which is located on chromosome 16q12.2 and has nine exons, is strongly conserved across various vertebrate species (for example, fish and chicken) and emerged 450 million years ago [7]. *FTO *is mainly expressed in the hypothalamus and encodes a 2-oxoglutarate-dependent nucleic acid demethylase. It may play important roles in the management of energy homeostasis [7,8], nucleic acid demethylation, and the regulation of body fat masses by lipolysis [9].

The *FTO *gene has recently attracted much attention in obesity research. Previous genetic association-based studies have shown that SNPs in the *FTO *gene are associated with increased body mass index (BMI) [5,10,11], and/or other metabolic-related traits, such as higher fasting insulin [12], glucose [12], triglycerides [12], lower HDL cholesterol [12], waist circumference [11,13], and weight [5]. For example, Scuteri *et al*. [5] showed that SNP rs9930506 within *FTO *was associated with BMI, total body weight, and hip circumference; many variants have been reported to be associated with obesity, including rs9939609, rs1421085, rs8050136, rs17817449, and rs1121980. These single nucleotide polymorphisms (SNPs) lie within a 47-kilobase linkage disequilibrium (LD) block encompassing parts of the first two introns and exon 2 of *FTO*. The transcriptional start site of the retinitis pigmentosa GTPase regulator-interacting protein 1-like (RPGRIP1L) gene is also near the 5' end of *FTO *[4]. Based on this observation, Fawcett *et al*. [14] argued that the association signal could be due to a correlation between *FTO *intronic SNPs and RPGRIP1L.

Of the SNPs that were reported to associate with obesity, rs9939609 has been of particular interest since it was discovered by Frayling *et al*. [4] The associations of other SNPs in the *FTO *gene with obesity (or overweight) have been replicated in large European populations [5,6]. In some different ethnic groups, however, such as the Chinese [15] and Oceanic populations [16], no association was observed between rs9939609 and obesity by either genetic association studies or GWAS. However, as more studies are reported, particularly with respect to various ethnicities, there is a need to determine more precisely the effect sizes in each major racial group and to investigate the minor allele frequency (MAF) and the LD patterns of the SNPs across different ethnicities. In addition, some reports have claimed ethnic-specific associations with alternative SNPs, and to that end there has been a degree of confusion. Therefore, we conducted meta-analyses of all available data.

## Methods

### Publication search

We searched PubMed, MEDLINE, Web of Science, EMBASE, and BIOSIS Preview up to October 2010 for studies concerning the association between *FTO *polymorphisms and obesity (or obesity-related traits including body weight, leptin levels, subcutaneous fat, fat mass, hip and waist circumference, lean mass, body height, and BMI). There are no limits on language. Two search themes were combined using the Boolean operator "and". The first theme was ("*obesity*" (Mesh) OR "*overweight*" (Mesh) OR "*body mass index" *(Mesh) OR "*obesity, morbid*" (Mesh) OR "*morbid obesity*" OR "*morbid obesities*" OR "*BMI*" OR "*body weight*" OR "*leptin levels*" OR "*subcutaneous fatness*" OR "*fat mass*" OR "*hip circumference*" OR "*waist circumference*" OR "*lean mass*" OR "*body height*"). The second theme was ("*rs9939609*" OR "*rs8050136*" OR "*rs1421085*" OR "*rs17817449*" OR "*rs1121980*" OR "*FTO*" OR "*FTO protein, human*" (Substance Name) OR "*fat mass and obesity associated*" (Substance Name)). Meta-analysis articles were not excluded because several original studies were often combined in meta-analysis articles on genetic association studies.

### Selection

Genetic association studies and GWASs with case-control subjects in which the case subjects were obese and the control subjects were healthy were included. At least two studies had to be available for each SNP. Studies were excluded if the control subjects violated the Hardy-Weinberg Equilibrium (HWE).

### Data extraction

Two reviewers (SHP and YMZ) independently searched the databases. The search results were then evaluated by five reviewers (SHP, YMZ, FYX, XBR, and XBL). Disagreements were resolved by discussions among the reviewers. Information on gender, author name, country, ethnicity, year of publication, mean age of examination, mean BMI (calculated as weight in kilograms divided by height in meters squared), and genotypes (for example, TT/TA/AA) were retrieved. MAF and *P*-values of the HWE were calculated from the above genotype data. Adult individuals with BMI ≥30 kg/m^2 ^were defined as case subjects of obesity, and individuals with BMI <30 kg/m^2 ^were considered control subjects. The criteria for childhood obesity described by Cole *et al*. [17] were adopted.

### Statistical analysis

To maximize the number of studies included, analyses were conducted by calculating the ORs under per-allele comparison. We found that an additive genetic model was always employed in most of the genetic association studies concerning the association between *FTO *and obesity risk. Therefore, the overall effects were also calculated in an additive genetic model in this meta-analysis. For comparison, the ORs in dominant and recessive genetic models were also calculated. Recently, Zintzaras [18] reported a novel method to calculate the generalized odds ratio (ORG), which may overcome the shortcomings of multiple model testing or erroneous model specification. Thus, the ORG calculations were also performed.

All statistical tests were performed with Stata 10.0 software (Stata, College Station, TX, USA). The effect sizes were calculated by the DerSimonian-Laird method, which is a random effect model because a modest or higher heterogeneous status was found in both allelic comparison and additive genetic models.

The Cochran's χ^2 ^test (Q test) was used to evaluate heterogeneity between studies, and a threshold *P*-value of 0.1 was considered statistically significant. The *I*^2 ^test was also conducted to evaluate heterogeneity between studies [19]. Begg's and Egger's tests were both used to test the significance of the publication bias, with a *P*-value < 0.1 considered as significant. We used the software developed by Guo *et al*. [20] to test (using exact test [21]) the significance of the HWE in control subjects, with a threshold *P*-value of 0.05 and 0.001 considered statistically significant for candidate gene association studies and GWAS [22,23], respectively. Heterogeneity is considered higher when *I*^2 ^>50% and much higher when *I*^2 ^>75% [24], and higher heterogeneity is a common phenomenon in genetic association studies [25,26]. To estimate heterogeneity, meta-regression was performed with the mean age of control subjects (Age_Control) and the mean BMI of control subjects (BMI_Control) as the covariates [27].

### Analyses of sensitivity, subgroup, and LD pattern

To identify the source of the heterogeneity between studies, we performed sensitivity analyses by including and excluding some studies. Sensitivity analyses were done sequentially for all of the SNPs and all of the studies (or by some subgroups of the studies).

We sub-grouped the studies into six groups (Caucasian, Asian, Hispanic, African, South American, and Mixed) on rs9939609, four groups (Caucasian, Asian, African, and Mixed) on rs8050136, and two groups (Caucasian, Asian) on rs1121980.

The LD patterns of the SNPs were investigated using the HapMap Database (http://hapmap.ncbi.nlm.nih.gov/) and HaploView software (Whitehead Institute for Biomedical Research, Cambridge, USA) [28].

## Results

### Characteristics of the included studies

**Figure 1:**
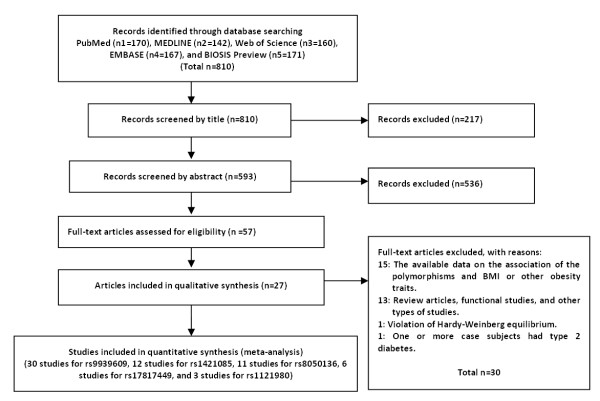
Flow diagram of the study selection for the meta-analyses.

According to the search strategy, 170, 142, 160, 167, and 171 articles (a total of 810 articles) were retrieved from PubMed, MEDLINE, Web of Science, EMBASE, and BIOSIS Preview, respectively. After the first screening, in which the abstracts or titles were read, 753 articles were excluded, and 59 articles underwent further review. After reviewing these articles, 30 additional articles were excluded, which left a total of 27 articles for inclusion in this meta-analysis. According to the PRISMA guidelines [29], the flow diagram is shown in Figure 1. A total of 21 articles [4,15,30-48] included 30 studies on rs9939609; five articles [6,33,41,49,50] included eleven studies on rs1421085; seven articles [15,30,31,43,46,51,52] included nine studies on; rs8050136, two articles [6,33] included six studies on rs17817449; and three articles [37,43,53] included three studies on rs1121980. Not counting the overlapping literature, 27 articles were obtained (Figure 1). In the 30 studies concerning rs9939609, subgroup analyses were performed. The 30 studies were divided into six subgroups according to ethnicity, as follows: studies with Caucasian populations [4,33,36,41-45,47], Asian populations [15,31,32,35,37,46,48], Hispanic groups [31,38,39], South American [40], African [31], and studies using mixed ethnic populations [34]. In all of the included studies, the genotype distributions in the control subjects are consistent with the HWE. Three studies by Song *et al*. [31] (concerning rs9939609 and rs8050136) and one study by Price *et al*. [33] were excluded due to the deviation from the HWE (with a *P*-value of less than 0.05).

Unfortunately, some articles were excluded because the associations were between one or more SNPs and obesity-related traits rather than obesity itself (for example, ref. [5,10,11,16,54,55]). There are five included articles [15,34,51-53] with OR values and other detailed information but without genotype data. We attempted to obtain the genotyped data from these studies by e-mail, but we received no response from the authors.

The clinical characteristics of the included studies are shown in Table 1. The genotype data, as well as the ORs under both per-allele comparisons and the additive genetic model, are shown in Table 2. The ORs under dominant and recessive genetic models and the OR_G _are shown in Table S1 (see Additional file 1). These studies were published between 2007 and 2010. A total of 59 studies relating to all five SNPs were included, involving a total of 41,734 obesity cases and 69,837 healthy controls.

**Table 1:** Clinical characteristics of the included studies

Study	Publication year	Country	Ethnicity	Case		Control	
				
				Mean age (SD) (years)	Mean BMI (SD) (*kg/m^2^*)	Mean age (SD) (years)	Mean BMI (SD) (*kg/m^2^*)
Frayling_a [4]*^a ^*	2007	UK	Caucasian	NA*^g^*	NA	28.4 (4.7)	22.7
Frayling_b	2007	UK	Caucasian	NA	NA	31.0	24.4
Frayling_c	2007	UK	Caucasian	NA	NA	40.6 (6.1)	25.6
Frayling_d	2007	UK	Caucasian	NA	NA	56.7 (4.5)	26.4
Frayling_e	2007	UK	Caucasian	60.1 (8.8)	32.8	59.7 (9.0)	26.1
Frayling_f	2007	UK	Caucasian	NA	NA	68.8 (5.5)	27.2
Frayling_g	2007	UK	Caucasian	NA	NA	74.3 (6.9)	27.2
Hinney [43]*^b ^*	2007	Germany	Caucasian	14.4 (3.7)	33.4 (6.8)	26.1 (5.8)	18.3 (1.1)
Dina_a [6]	2007	France	Caucasian	44 (12)	47 (7.6)	51 (10)	22.8 (2)
Dina_b	2007	France	Caucasian	10.6 (3.5)	NA	49 (5.5)	22.3 (2.3)
Dina_c	2007	France	Caucasian	11.8 (3.1)	29.8 (5.8)	22.0 (3.7)	21.1 (2.0)
Dina_d	2007	Switzerland	Caucasian	NA	46.7 (5.5)	NA	NA
Dina_e	2007	Germany	Caucasian	11.6 (2.7)	NA	11.7 (1.7)	NA
Song_H [31]*^c ^*	2007	USA	Hispanic	NA	NA	61.1 (6.71)	30.9 (7.1)
Song_AP	2007	USA	Asian	NA	NA	60.2 (6.77)	29.0 (5.78)
Song_B	2007	USA	African	NA	NA	63.8 (7.58)	24.7 (4.49)
Villalobos [40]	2008	Mexico	South American	43.9 (13.1)	39.4 (8.6)	NA	NA
Chang [35]	2008	China-Taiwan	Asian	37.0 (0.56)	38.9 (8.2)	61.1 (0.33)	24.0 (2.9)
Hotta [37]	2008	Japan	Asian	49.1 (14.2)	34.5 (5.4)	48.2 (16.5)	21.7 (2.1)
Li [15]	2008	China	Asian	NA	NA	58.6 (6.0)	24.2
Peeters [41]	2008	Belgium	Caucasian	NA	38.3 (0.3)	NA	NA
Jacobsson_2008 [42]	2008	Sweden	Caucasian	12.6 (3.3)	35.4 (6.6)	17.1 (0.8)	21.1 (2.6)
Muler [44]*^d^*	2008	Germany	Caucasian	10.7 (3.1)	28.9 (5.2)	24.6 (2.6)	21.8 (1.1)
Andreasen_a [45]	2008	Denmark	Caucasian	NA	NA	46.2 (7.9)	26.3 (4.6)
Andreasen_b*^e^*	2008	Denmark	Caucasian	NA	NA	60.0 (6.8)	28.6 (4.9)
Andreasen_c	2008	Denmark	Caucasian	NA	NA	58.5 (8.1)	26.5 (4.2)
Grant_C [51]	2008	USA	Caucasian	2-18	BMI ≥95th percentile	2-18	BMI<95th percentile
Grant_AA	2008	USA	African	2-18	BMI ≥95th percentile	2-18	BMI<95th percentile
Price [33]	2008	USA	Caucasian	41.1 (9.4)	49.1 (8.8)	42.6 (8.8)	20.8 (1.8)
Legry [36]	2008	France	Caucasian	52.9 (8.2)	33.8 (3.5)	48.9 (8.5)	22.5 (1.8)
Gonzalez [39]	2009	Spain	Hispanic	58.0 (11.0)	33.0 (3.3)	54.0 (11.0)	25.8 (2.6)
Zabena [38]	2009	Spain	Hispanic	44.8 (12.5)	45.5 (7.4)	45.5 (7.3)	24.3 (2.4)
Tabara [32]*^f^*	2009	Japan	Asian	NA	NA	61.0 (14.0)	23.4 (3.2)
Attaoua [49]	2009	France	Caucasian	39.1 (11.4)	39.4 (3.8)	39.6 (11.6)	23.8 (2.6)
Meyre_FC [50]	2009	France	Caucasian	11.5 (3.1)	29.0 (9.8)	21.0 (4.5)	20.74 (2.2)
Meyre_FA	2009	France	Caucasian	41.1 (10.9)	49.4 (7.8)	49.1 (5.5)	22.3 (2.3)
Meyre_SA	2009	Switzerland	Caucasian	42.4 (11.5)	43.7 (7.2)	NA	NA
Meyre_GC	2009	Germany	Caucasian	11.8 (2.9)	29.7 (9.2)	11.8 (2.9)	18.15 (8.1)
Thorleifsson [52]	2009	Mixed	Mixed	NA	NA	NA	NA
Willer [34]	2009	Mixed	Mixed	NA	NA	NA	NA
Renstrom [53]	2009	Sweden	Caucasian	52.6 (9.5)*^h ^*	>30	52.6 (9.5) *^h^*	18.5-24.9
Jacobsson_2009 [47]	2009	Sweden	Caucasian	50.0 (0)	32.1 (1.9)	50.0 (0)	24.8 (1.8)
Karasawa [48]*^f ^*	2010	Japan	Asian	NA	NA	63.0 (1.2)	23.5 (3.2)
Liu [46]	2010	China	Asian	NA	NA	58.0 (9.0)	24.5 (3.2)
Cheung [30]	2010	China	Asian	46.3 (11.9)	32.2 (4.9)	45.0 (12.5)	21.2 (1.2)

*^a^*: The article by Frayling *et al*. contains seven studies, namely Frayling _a (ALSPAC), Frayling _b (NFBC1966), Frayling _c (Oxford Biobank), Frayling _d (Caerphilly), Frayling _e (EPIC-Norfolk), Frayling _f (BWHHS), and Frayling _g (InCHIANTI). Please refer to the original article [4] for details.

*^b^*: Early-onset, extremely obese cases (mean BMI Z score 4.63 ± 2.27) and healthy, underweight controls (mean BMI Z score -1.38 ± 0.35; BMI <15^th^age percentile).

*^c^*: The age and BMI values are reported as the mean value of all case and control subjects.

*^d^*: Obese cases are reported as the 99.5^th ^percentile of the median age and gender-specific BMI. Lean controls are reported as the 51.0^st ^percentile of the median age and gender-specific BMI.

*^e^*: The article by Andreasen *et al*. contains three studies, including Andreasen_a (Population-based, Inter99 study sample), Andreasen_b

(ADDITION study cohort), and Andreasen_c (SDC control group). For details, please refer to the original article [45].

*^f^*: In Japan, obesity is defined as a condition in which the individuals had a BMI ≥25 kg/m^2 ^according to the guidelines of the Japanese Society for the Study of obesity [68].

*^g^*: Not available.

*^h^*: The mean age of all subjects (non-diabetic individuals).

**Table 2:** Genotypic distributions and ORs for the association between five polymorphisms and obesity

Study	N (case/control)						OR [95% CI], *P*-value	
	
	Subtotal	MAF	*P*HWE	**11***^a^*	12	22	Additive model	Allelic comparison
**rs9939609**								

Caucasian:								
Frayling_a	5,380 (353/5,027)	0.48/0.39	0.513	107/1,879	157/2,407	89/741	1.43 (1.23 to 1.67)	1.43 (1.23 to 1.67)
Frayling_b	3,072 (415/2,657)	0.45/0.37	0.185	132/1,060	193/1,211	90/386	1.36 (1.18 to 1.57)	1.37 (1.18 to 1.59)
Frayling_c	487 (126/361)	0.42/0.38	0.575	45/138	56/175	25/48	1.21 (0.90 to 1.62)	1.21 (0.91 to 1.61)
Frayling_d	623 (200/423)	0.41/0.34	0.447	67/189	101/182	32/52	1.37 (1.07 to 1.75)	1.37 (1.08 to 1.76)
Frayling_e	2,511 (1,588/923)	0.43/0.38	0.625	485/351	847/443	256/129	1.24 (1.10 to 1.40)	1.22 (1.09 to 1.37)
Frayling_f	1,875 (840/1,035)	0.42/0.37	0.547	274/421	427/471	139/143	1.26 (1.10 to 1.44)	1.25 (1.10 to 1.43)
Frayling_g	461 (212/249)	0.47/0.42	0.514	66/82	92/127	54/40	1.25 (0.97 to 1.61)	1.26 (0.97 to 1.63)
Andreasen_a	3,724 (1,045/2,679)	0.45/0.39	0.103	313/993	517/1,309	215/377	1.33 (1.20 to 1.48)	1.32 (1.19 to 1.47)
Andreasen_b	4,671 (2,819/1,852)	0.43/0.39	0.221	911/684	1,385/904	523/264	1.21 (1.11 to 1.31)	1.20 (1.11 to 1.31)
Andreasen_c	377 (104/273)	0.45/0.39	0.076	30/93	55/145	19/35	1.27(0.91 to 1.80)	1.25 (0.90 to 1.72)
Muler	697 (519/178)	0.50/0.44	0.763	140/56	238/86	141/36	1.24 (0.98 to 1.57)	1.26 (0.99 to 1.60)
Price	1,047 (527/520)	0.48/0.36	0.084	152/207	242/257	133/56	1.69 (1.41 to 2.02)	1.69 (1.42 to 2.02)
Peeters	1,367 (1,099/268)	0.46/0.40	0.703	316/94	553/133	230/41	1.28 (1.05 to 1.56)	1.28 (1.05 to 1.55)
Jacobsson_2008	960 (450/510)	0.48/0.42	0.716	133/174	206/244	111/92	1.24 (1.04 to 1.48)	1.25 (1.05 to 1.50)
Legry	3,367 (598/2,769)	0.46/0.41	0.050	171/924	307/1,395	120/450	1.20 (1.05 to 1.36)	1.19 (1.05 to 1.35)
Jacobsson_2009	1,115 (54/1,061)	0.44/0.39	0.218	16/411	29/483	9/167	1.22 (0.83 to 1.80)	1.23 (0.83 to 1.82)
Hinney	926 (484/442)	0.50/0.39	0.690	123/164	241/214	120/64	1.57 (1.30 to 1.90)	1.57 (1.30 to 1.88)
Asian:								
Song_AP	240 (77/163)	0.20/0.17	0.409	50/114	23/43	4/6	1.23 (0.76 to 1.97)	1.24 (0.76 to 2.03)
Chang	2,135 (610/1,525)	0.17/0.13	0.355	425/1,158	167/347	18/20	1.38 (1.15 to 1.66)	1.37 (1.14 to 1.5)
Hotta	2,423 (919/1,504)	0.24/0.19	0.393	534/1,005	334/443	51/56	1.37 (1.19 to 1.58)	1.66 (1.31 to 2.11)
Tabara	1,718 (214/1,504)	0.22/0.16	0.438	128/1,063	77/408	9/33	1.54 (1.20 to 1.99)	1.53 (1.19 to 1.95)
Karasawa	2,639 (794/1,845)	0.23/0.20	0.373	477/1,203	271/566	46/76	1.22 (1.06 to 1.41)	1.23 (1.06 to 1.42)
Liu	1,167 (276/891)	0.14/0.11	0.396	201/701	70/181	5/8	1.38 (1.04 to 1.83)	1.36 (1.03 to 1.80)
Li	3,210 (398/2,812)	NA/0.12	NA	NA	NA	NA	0.90 (0.70 to 1.15)	NA
Hispanic:								
Song_H	415 (139/276)	0.27/0.31	0.157	75/138	53/107	11/31	0.85 (0.62 to 1.16)	0.84 (0.61 to 1.15
Gonzalez	725 (207/518)	0.45/0.37	0.925	62/203	104/244	41/71	1.38 (1.09 to 1.74)	1.37 (1.09 to 1.73)
Zabena	255 (75/180)	0.52/0.34	1.0	19/78	34/81	22/21	2.04 (1.38 to 3.02)	2.09 (1.42 to 3.07)
African:								
Song_B	1,114 (365/749)	0.47/0.45	0.160	113/239	161/351	91/159	1.09 (0.92 to 1.30)	1.10 (0.92 to 1.31)
South American:								
Villalobos	788 (364/424)	0.25/0.17	0.598	209/297	127/114	28/13	1.66 (1.31 to 2.11)	1.7 (1.33 to 2.17)
Mixed:								
Willer	19,367 (5,261/14,106)	NA	NA	NA	NA	NA	NA	1.25 (1.19 to 1.31)

**Total N (rs9939609)**	68,856 (21,132/47,724)					Pooled ORs	**1.31 (1.25 to 1.37)**	**1.31 (1.26 to 1.36)**

**rs1421085**								

Causian:								
Dina_a	3,557 (867/2,690)	0.52/0.41	0.218	200/944	425/1,273	242/473	1.55 (1.39 to 1.73)	1.57 (1.41 to 1.75)
Dina_b	1,678 (699/979)	0.51/0.42	0.896	173/323	334/481	192/175	1.42 (1.24 to 1.63)	1.43 (1.25 to 1.64)
Dina_c	1,001 (482/519)	0.51/0.41	0.205	119/187	233/237	130/95	1.47 (1.24 to 1.75)	1.50 (1.26 to 1.79)
Dina_d	1,018 (504/514)	0.52/0.46	0.051	123/161	235/233	146/120	1.26 (1.07 to 1.49)	1.29 (1.08 to 1.53)
Dina_e	551 (283/268)	0.53/0.40	0.637	62/246	142/343	79/110	1.69 (1.38 to 2.06)	1.67 (1.37 to 2.04)
Peeters	1,367 (1,099/268)	0.48/0.41	0.900	300/93	544/129	255/46	1.31 (1.08 to 1.59)	1.31 (1.09 to 1.59)
Attaoua	248 (119/129)	0.55/0.41	0.585	24/47	58/59	37/23	1.78 (1.24 to 2.54)	1.81 (1.27 to 2.59)
Meyre_FC	1,036 (491/545)	0.49/0.41	0.077	137/197	231/244	123/104	1.31 (1.11 to 1.55)	1.33 (1.12 to 1.59)
Meyre_FA	859 (132/727)	0.51/0.43	0.820	31/238	67/359	34/130	1.42 (1.09 to 1.85)	1.41 (1.09 to 1.84)
Meyre_SA	1,346 (1,028/318)	0.50/0.46	0.055	286/100	460/141	282/77	1.13 (0.96 to 1.34)	1.15 (0.96 to 1.37)
Meyre_GC	1,080 (370/710)	0.55/0.45	0.081	76/227	181/328	113/155	1.47 (1.23 to 1.75)	1.50 (1.25 to 1.79)

**Total N (rs1421085)**	13,741 (6,074/7,667)					Pooled ORs	**1.41 (1.31 to 1.51)**	**1.43 (1.33 to 1.53)**

**rs8050136**								

Caucasian:								
Hinney	928 (486/442)	0.50/0.39	0.842	123/165	240/212	123/65	1.58 (1.31 to 1.91)	1.59 (1.32 to 1.91)
Grant_C	2,688 (418/2,270)	0.45/0.39	NA	NA	NA	NA	NA	1.27 (1.09 to 1.47)
Asian:								
Song_AP	240 (77/163)	0.20/0.18	0.425	52/111	20/45	5/7	1.09 (0.68 to 1.73)	1.10 (0.67 to 1.78)
Cheung	1,159 (468/691)	0.16/0.12	0.463	326/535	131/149	11/7	1.48 (1.16 to 1.89)	1.46 (1.15 to 1.85)
Liu	1,180 (278/902)	0.15/0.11	0.489	201/716	73/178	4/8	1.43 (1.08 to 1.91)	1.42 (1.07 to 1.87)
Li	3,210 (398/2,812)	NA/0.12	NA	NA	NA	NA	0.91 (0.71 to 1.16)	NA
African:								
Song_B	1,114 (365/749)	0.42/0.42	0.454	133/257	161/355	71/137	0.98 (0.82 to 1.17)	0.98 (0.82 to 1.17)
Grant_AA	2,002 (578/1,424)	0.45/0.44	NA	NA	NA	NA	NA	1.05 (0.91 to 1.21)
Mixed:								
Thorleifsson	22,277 (13,785/8,492)	NA	NA	NA	NA	NA	NA	1.27 (1.21 to 1.32)

**Total N **(rs8050136)	34,798 (16,853/17,945)					Pooled ORs	**1.22 (0.99 to 1.51)**	**1.25 (1.13 to 1.38)**

**rs17817449**								

Caucasian:								
Dina_a [6]	3,548 (873/2,675)	0.49/0.60	0.968	212/948	426/1,288	235/439	1.55 (1.39 to 1.72)	1.55 (1.39 to 1.73)
Dina_b	1,589 (689/900)	0.47/0.59	0.600	164/337	320/489	205/164	1.59 (1.38 to 1.83)	1.60 (1.40 to 1.84)
Dina_c	1,010 (485/525)	0.49/0.60	0.173	120/196	236/237	129/92	1.52 (1.28 to 1.81)	1.55 (1.30 to 1.85)
Dina_d	1,035 (516/519)	0.50/0.55	0.374	138/164	243/246	135/109	1.21 (1.02 to 1.43)	1.22 (1.03 to 1.45)
Dina_e	972 (281/691)	0.46/0.58	0.755	58/231	142/341	81/119	1.65 (1.35 to 2.01)	1.63 (1.34 to 1.99)
Price	1,049 (527/522)	0.49/0.36	0.087	145/205	247/259	135/58	1.72 (1.44 to 2.06)	1.72 (1.44 to 2.05)

**Total N (rs17817449)**	9,203 (3,371/5,832)					Pooled ORs	**1.53 (1.40 to 1.67)**	**1.54 (1.41 to 1.68)**

**rs1121980**								

Caucasian:								
Hinney	926 (484/442)	0.53/0.41	0.694	104/153	247/218	133/71	1.66 (1.37 to 2.01)	1.64 (1.37 to 1.97)
Renstrom	1,723 (353/1,370)	NA	NA	NA	NA	NA	NA	1.15 (1.05 to 1.25)
Asian:								
Hotta	2,451 (927/1,524)	0.26/0.21	0.593	499/947	367/504	61/73	1.32 (1.15 to 1.51)	1.32 (1.16 to 1.51)

**Total N **(**rs1121980)**	5,100 (1,764/3,336)					Pooled ORs	**1.46 (1.17 to 1.83)**	**1.34 (1.10 to 1.62)**

**Total Subjects (duplicated subjects were excluded)**							112,327 (42,616/69,711)	

*^a^*: 11, 12, and 22 denote TT, TA, and AA for rs9939609; TT, TC, and CC for rs1421085; CC, CA, and AA for rs8050136; TT, TG, and GG for rs17817449; and GG, GA, and AA for rs1121980.

We estimated the MAF in the five polymorphisms from the control subjects of all studies identified for inclusion in the present meta-analysis. Across all studies, the MAFs ranged between 11% and 45%, 40% and 46%, 11% and 44%, 36% and 60%, and 21% and 44% for rs9939609, rs1421085, rs8050136, rs17817449, and rs1121980, respectively.

### Meta-analysis of FTO gene SNP rs9939609

**Figure 2:**
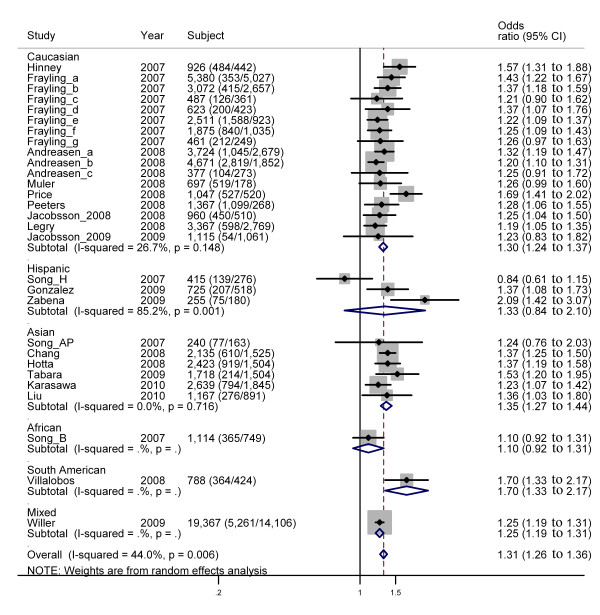
**Forest plot of the association between obesity and the rs9939609 polymorphism under per-allele comparison**. On the left, the name of the first author of the study is followed by the publication year in parentheses. The size of the black box corresponding to each study is proportional to the sample size, and the horizontal line shows the corresponding 95% CI of the odds ratio (OR). The overall ORs are based on a random-effects model shown by the diamonds. The solid, vertical line represents the null result.

Under per-allele comparison, the OR is not available from the study by Li *et al*., leaving 29 studies for further consideration. A total of 21 out of 29 studies reported a significant, positive association between obesity and the rs9939609 genotype (Table 2, Figure 2). A significant association between obesity and rs9939609 was detected, with an overall OR of 1.31 (95% CI: 1.26 to 1.36) under per-allele comparison, and there is evidence of heterogeneity (*I*^2 ^= 44.0%). The Begg's test (*P *= 0.39), and Egger's test (*P *= 0.17) provided no evidence of publication bias.

When stratifying the data by ethnicity, no evidence of a significant association was observed in the three studies with Hispanic ethnic groups (summary OR: 1.33, 95% CI: 0.84 to 2.10) under per-allele comparison, with the largest heterogeneity in all six ethnic groups (*I*^2 ^= 85.2%, *P *= 0.001) (Figure 2). We observed overall ORs of 1.30 (95% CI: 1.24 to 1.37) and 1.35 (95% CI: 1.27 to 1.44) in the Caucasian and Asian ethnic groups for which more studies had been performed under per-allele comparison.

By using meta-regression, we detected a significant correlation between the mean control BMI and effect size in an allelic comparison (*P *= 0.03) (see Additional file 1: Figure S1).

Very similar results were obtained using an additive genetic model, with an overall OR of 1.31 (95% CI: 1.25 to 1.37) and evidence of heterogeneity (*I*^2 ^= 52.8%) (Table 2, Additional file 1: Figure S2).

### Meta-analysis of FTO SNPs rs1421085, rs8050136, s17817449, and rs1121980

#### rs1421085

**Figure 3:**
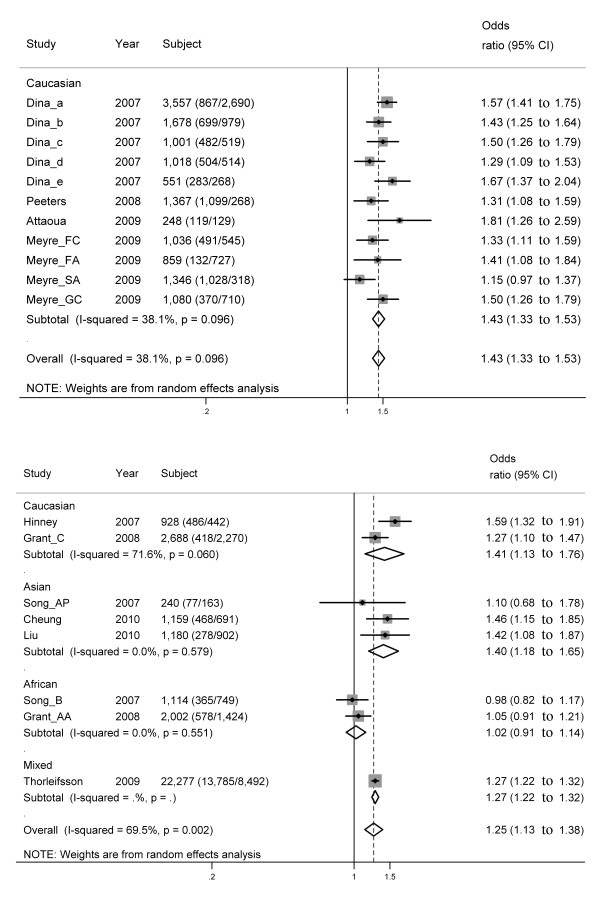
**Forest plot of the association between obesity and the two SNPs under per-allele comparison**. On the left, the name of the first author of the study is followed by the publication year in parentheses. The size of the black box corresponding to each study is proportional to the sample size, and the horizontal line shows the corresponding 95% CI of the odds ratio (OR). The overall ORs are based on a random-effects model shown by the diamonds. The solid, vertical line represents the null result. (**A**) rs1421085; (**B**) rs8050136.

A total of 11 out of 12 studies reported a significant, positive association between obesity and the rs1421085 genotype (Table 2, Figure 3A). Also under per-allele comparison, a significant association between obesity risk and rs1421085 was found (overall OR = 1.43, 95% CI: 1.33 to 1.53), and there is evidence of heterogeneity (*I*^2 ^= 38.1%). Begg's test (*P *= 0.76) and Egger's test (*P *= 0.84) provided no evidence of publication bias.

By using meta-regression, no significant correlation was found between the mean control BMI and effect size in an allelic comparison (*P *= 0.50).

#### rs8050136

Overall, 11 studies have investigated the association of rs8050136 with obesity. Of the 11 studies, the OR (under per-allele comparison) of the study by Li *et al*. is not available. Of the remaining 10 studies, 5 reported a significant, positive association between obesity and the rs8050136 genotype (Table 2, Figure 3B). A significant association between obesity risk and rs8050136 was found (overall OR = 1.25, 95% CI: 1.13 to 1.38) under per-allele comparison, and there is evidence of higher heterogeneity (*I*^2 ^= 72.0%). Begg's test (*P *= 1.0) and Egger's test (*P *= 0.87) provided no evidence of publication bias.

By using meta-regression, no significant correlation was found between the mean control BMI and effect size in an allelic comparison (*P *= 0.60).

#### rs17817449

**Figure 4:**
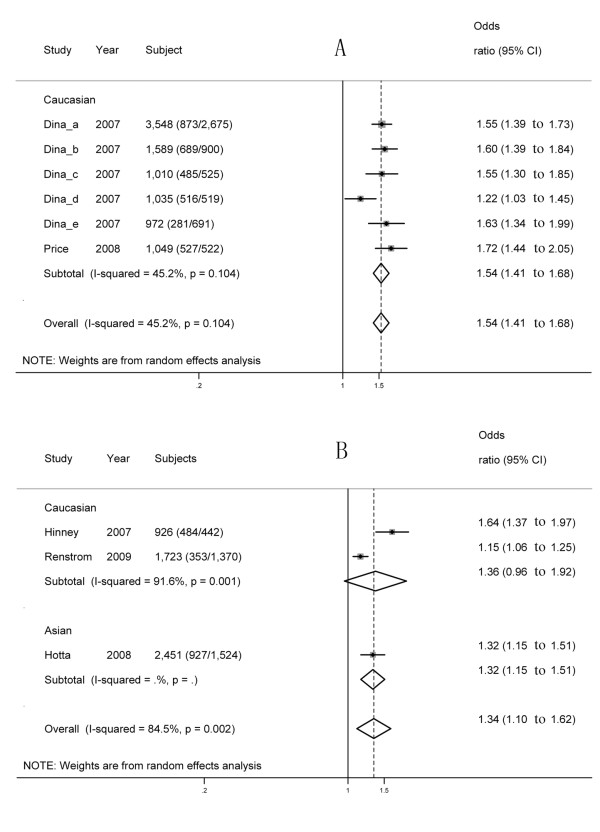
**Forest plot of the association between obesity and the two SNPs under per-allele comparison**. On the left, the name of the first author of the study is followed by the publication year in parentheses. The size of the black box corresponding to each study is proportional to the sample size, and the horizontal line shows the corresponding 95% CI of the odds ratio (OR). The overall ORs are based on a random-effects model shown by the diamonds. The solid, vertical line represents the null result. **(A) **rs17917449; **(B) **rs1121980.

All six of the studies involving rs17817449 reported a significant, positive association between obesity and rs17817449 (Table 2, Figure 4A). A significant association between obesity risk and rs17817449 was found (overall OR = 1.54, 95% CI: 1.41 to 1.68) for obesity and rs17817449 under per-allele comparison, and there is evidence of higher heterogeneity (*I*^2 ^= 45.2%). Begg's test (*P *= 0.85) and Egger's test (*P *= 0.90) provided no evidence of publication bias.

By using meta-regression, no significant correlation was found between the mean control BMI and effect size in an allelic comparison (*P *= 0.57).

#### rs1121980

All three studies reported a significant, positive association between obesity and the rs1121980 SNP (Table 2, Figure 4B). A significant association between obesity risk and rs1121980 was found (overall OR = 1.34, 95% CI: 1.10 to 1.62) under per-allele comparison, with evidence of greater heterogeneity (*I*^2 ^= 84.5%). Begg's test (*P *= 0.12) provided no evidence of publication bias, whereas the funnel plot (not shown) and Egger's test (*P *= 0.09) showed evidence of publication bias.

By using meta-regression, no significant correlation was found between the mean control BMI and effect size in an allelic comparison (*P *= 0.28).

## Discussion

To our knowledge, this meta-analysis is the first one to investigate the associations between *FTO *polymorphisms and obesity risk across different ethnic groups. This meta-analysis investigated the associations between five *FTO *polymorphisms (rs9939609, rs1421085, rs8050136, rs17817449, and rs1121980) and obesity risk in 41,734 cases and 69,837 controls from 59 studies, counting the cases and control subjects from each study only once. We found significant evidence for a modest increase in the risk of obesity associated with the five polymorphisms in various ethnic populations (Figures 2, 3, 4). However, subgroup analyses showed that in some ethnic populations, for example, rs9939609 in Hispanic and African, rs1121980 in Caucasian, and rs8050136 in Asian and African, significant associations were not found between the SNPs and obesity risk (see Additional file 1: Figure S3). These results may be due to the differences in MAF or LD patterns across different ethnic populations.

Interestingly, we found that MAF values are very similar across all five SNPs. For example, the MAFs in the study by Price *et al*. are 0.36 for rs9939609 and 0.36 for rs17817449. There are also ethnic variations of the MAF in the five polymorphisms. Caucasian, US Hispanics, and US Africans have higher MAFs (>31%), whereas Asian populations (for example, Chinese and Japanese) and South Americans have lower MAFs (between 11% and 20%). When stratifying the studies on rs9939609 into subgroups, the MAFs are different in various populations. For example, the MAF in control subjects is 0.34 to 0.44 in Caucasians, 0.11 to 0.20 in Asians, 0.31 to 0.37 in Hispanics, and 0.17 in South Americans, indicating that the MAF in Asian and South American populations is less than half of that in Caucasian and Hispanic populations. The population differences in MAF and LD structure probably have arisen through evolutionary divergence [56].

For rs9939609, we conducted sensitivity analyses. After the exclusion of the three children's studies [42-44], there is a small increase in overall heterogeneity (*I*^2 ^= 44.6%, *P *= 0.008 when exclusion of the three studies vs. *I*^2 ^= 44.0%, *P *= 0.006 when inclusion of all available studies) (see Additional file 1: Figure S4). Similarly, a slight increase in overall heterogeneity was observed after the exclusion of the two GWASs [4,43] (*I*^2 ^= 51.6%, *P *= 0.003 with the exclusion of the two studies vs. *I*^2 ^= 44.0%, *P *= 0.006 with the inclusion of all available studies). These results indicate that the children's studies or GWASs are not the sources of the heterogeneity. On the other hand, with the exclusion of the study by Price *et al*., the heterogeneity decreased rapidly (*I*^2 ^= 0.0%, *P *= 0.626 with the exclusion of the study vs. *I*^2 ^= 44.0%, *P *= 0.006 with the inclusion of all available studies) in the Caucasian subgroup, indicating that the study by Price *et al*. is the main source of heterogeneity in the Caucasian subgroup. By using an additive genetic model, coupled with either the inclusion or exclusion of the study by Li *et al*., the *I*^2 ^value in the heterogeneity test declined sharply (61.7% vs. 15.6%). Similarly, removing the study by Chang *et al*. caused the *I*^2 ^value to increase a little (61.7% vs. 67.0%), suggesting that the study by Li *et al*. is the main source of heterogeneity in the Asian ethnic group.

All of the studies on rs1421085 are from Caucasian populations, but when the five studies were excluded (two from Switzerland, two from Germany, and one from the USA) and the remaining seven studies were analyzed (six from France and one from Belgium), the *I*^2 ^decreased sharply from 38.1% to 0.0%. Similarly, the same tendency was found with rs17817449 (data not shown), suggesting that geographic stratification in an ethnic subgroup is one of the sources of the between-study heterogeneity.

**Figure 5:**
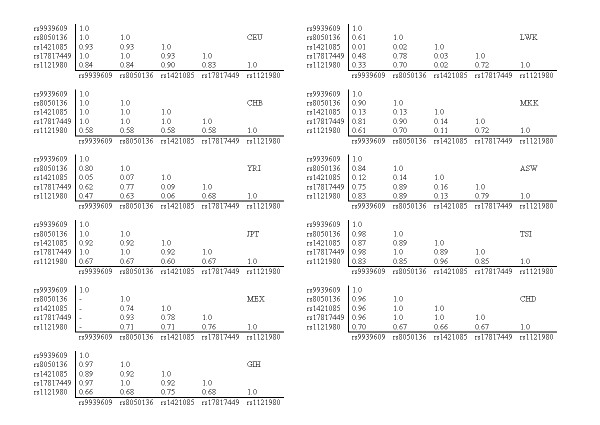
**LD pattern of the five SNPs obtained from HapMap database ASW**. African ancestry in Southwest USA; **CEU**, Utah residents with Northern and Western European ancestry from the CEPH collection; **CHB**, Han Chinese in Beijing, China; **CHD**, Chinese in Metropolitan Denver, Colorado; **GIH**, Gujarati Indians in Houston, Texas; **JPT**, Japanese in Tokyo, Japan; **LWK**, Luhya in Webuye, Kenya; **MEX**, Mexican ancestry in Los Angeles, California; **MKK**, Maasai in Kinyawa, Kenya; **TSI**, Tuscan in Italy; **YRI**, Yoruban in Ibadan, Nigeria.

We investigated the LD structure of the five SNPs in all available ethnic populations (Figure 5). We found that there are substantial differences in the LD structures in various ethnic populations. Similar ethnic populations showed similar patterns (Figure 5), including CEU with TSI; YRI, MKK, and LWK with ASW; and CHB and CHD with JPT. All five SNPs are in strong LD with each other in Caucasians (Figure 5). For example, in one Caucasian population, all five SNPs showed strong LD (*r*^2 ^≥ 0.83), and in this case, rs9939609 may be selected as a TagSNP for the other four SNPs. Similarly, in three different populations, including Caucasian, East Asian and Gujarati Indians, four of the five SNPs (rs9939609, rs8050136, rs1421085, and rs17817449) are also in strong LD, indicating that rs9939609 can be used as a TagSNP for the other three SNPs.

Although the five SNPs are in strong LD with each other in Caucasians, there are obvious differences in the effect size across the five SNPs: 1.3 (1.24 to 1.37) for rs9939609, 1.43 (1.33 to 1.53) for rs1421085, 1.41 (1.13 to 1.76) for rs8050136, 1.54 (1.41 to 1.68) for rs17819449, and 1.36 (0.96 to 1.92) for rs1121980 (Figures 2, 3, 4). The first four SNPs reported significant associations, and the last one reported non-significant associations. On the other hand, the five SNPs are not in strong LD across all the ethnic groups. Therefore, no SNP can be treated as tagSNP across all the ethnic groups. Considering these facts, we showed all the results concerning all the five SNPs without selection of tagSNPs. In fact, some studies with very small sample size were included in this meta-analysis, for example, only 240 subjects were included in the study by Song *et al*. (Song_AP, Table 2). This fact may be one of the reasons for the inconsistent results across the five SNPs in Caucasians. To address this issue, more studies with larger sample sizes are needed to provide more precise evidence.

The heterogeneities of all five SNPs are higher in the meta-analysis. There are many reasons for heterogeneity in genetic association studies, and these reasons can be divided into two categories: (1) differential biases due to population stratification, misclassification of clinical outcome, differences in BMI, genotyping errors and over-estimation of genetic effects in the first study and (2) differences in the pattern of LD structure across populations [57]. Using sensitivity analyses, we showed that population stratification may be the reason for higher heterogeneity in studies focused on rs1421085, indicating that population stratification can also exist within a subgroup of the same ethnic background (Caucasians). In the meta-regression analysis, a significant association was detected between the mean control BMI and effect size on rs9939609, suggesting that the difference in BMI value between the subjects may also be a source of heterogeneity.

Analyses of statistical power constitute a crucial step in the design of genetic association studies. Whereas conventional statistical power calculations for case-control studies disregard many basic elements of analytic complexity and can seriously underestimate true sample size requirements. Many methods have been proposed to address the sample size issue in genetic association study [58-60]. However, owing to many factors, such as financial pressure, difficulties in sample collection, or neglect of the importance of sample size, too few samples were employed in many genetic association studies, for example, Andreasen_c with total subjects of 377 and Song_AP with total subjects of 240.

### Limitations

Inherent limitations of this meta-analysis should be pointed out, including the following: (1) Based on the statistical tests, the heterogeneities are higher in the results of this meta-analysis. There may be many potential reasons for the higher heterogeneities, including differences in genetic susceptibility across ethnic groups and measurement error of BMI. (2) Gene-gene and gene-environment interactions could not be addressed in this meta-analysis. Actually, obesity is a complex trait, and many genes are related to obesity [61], such as *MC4R *[50,62,63], *MAF *[50], and *NPC1 *[50]. On the other hand, lifestyle factors, including diet and physical inactivity, are important contributors to weight gain and obesity [64-66] (3) It may be unreliable to use Begg's and Egger's test as the criteria for publication bias. Although publication bias was not found, it may exist due to the fact that some studies with non-significant associations were not published, and some articles published in other languages (not English) were not obtained and included in this meta-analysis [67]. (4) The Haplotype of the polymorphisms was not performed due to the lack of the related information.

## Conclusions

This meta-analysis suggests that FTO may represent a low-penetrance susceptible gene for obesity risk. Individual studies with large sample sizes are needed to further evaluate the associations between the polymorphisms and obesity risk in various ethnic populations.

## Abbreviations

BMI: body mass index; CI: confidence interval; GWAS: genome-wide association study; HWE: Hardy-Weinberg Equilibrium; LD: linkage disequilibrium; MAF: minor allele frequency; OR: odds ratio; ORG: generalized odds ratio; SNP: single nucleotide polymorphism.

## Competing interests

The authors declare that they have no competing interests.

## Authors' contributions

SHP, YMZ and MDL contributed to the design of the study. SHP, XBR, and XBL carried out the screening procedure. SHP, YMZ and FYX performed the statistical analysis. SHP drafted the manuscript. All authors read and approved the final manuscript.

## Pre-publication history

The pre-publication history for this paper can be accessed here:

http://www.biomedcentral.com/1741-7015/9/71/prepub

## Supplementary Material

Additional file 1 **Table S1, Figure S1, Figure S2, Figure S3, and Figure S4**. Click here for file
