# Supplementary material for: FTO gene polymorphisms and obesity risk: a meta-analysis
Source: BMC Med. 2011 Jun 8;9:71. doi: 10.1186/1741-7015-9-71 (PMC3118373; doi:10.1186/1741-7015-9-71)

# Supplementary material

**Table S1.** The ORs and 95% CI under the dominant and recessive genetic models, and the generalized odds ratio (ORG)

| **Study** | **OR (95% CI)** | | |
| --- | --- | --- | --- |
|  | **Dominant** ^a^ | **Recessive** ^b^ | **Generalized** ^c^ |
| rs9939609 | | | |
| Frayling_a | 1.37 (1.09 to 1.73) | 1.95 (1.51 to 2.51) | 1.49 (1.23 to 1.81) |
| Frayling_b | 1.42 (1.14 to 1.77) | 1.63 (1.26 to 2.11) | 1.43 (1.20 to 1.71) |
| Frayling_c | 1.11 (0.73 to 1.70) | 1.61 (0.95 to 2.75) | 1.22 (0.86 to 1.74) |
| Frayling_d | 1.60 (1.12 to 2.28) | 1.36 (0.84 to 2.19) | 1.48 (1.11 to 1.98) |
| Frayling_e | 1.40 (1.18 to 1.65) | 1.18 (0.94 to 1.49) | 1.29 (1.12 to 1.49) |
| Frayling_f | 1.42 (1.17 to 1.71) | 1.24 (0.96 to 1.59) | 1.32 (1.13 to 1.55) |
| Frayling_g | 1.09 (0.73 to 1.61) | 1.79 (1.13 to 2.82) | 1.28 (0.94 to 1.75) |
| Hinney | 1.73 (1.31 to 2.29) | 1.95 (1.39 to 2.72) | 1.71 (1.37 to 2.14) |
| Song_H | 0.85 (0.57 to 1.28) | 0.68 (0.33 to 1.40) | 0.83 (0.58 to 1.21) |
| Song_AP | 1.26 (0.71 to 2.23) | 1.43 (0.39 to 5.24) | 1.26 (0.72 to 2.18) |
| Song_B | 1.05 (0.80 to 1.37) | 1.23 (0.92 to 1.65) | 1.11 (0.90 to 1.37 |
| Villalobos | 1.73 (1.29 to 2.33) | 2.63 (1.34 to 5.17) | 1.74 (1.32 to 2.31) |
| Chang | 1.37 (1.11 to 1.69) | 2.29 (1.20 to 4.36) | 1.38 (1.13 to 1.70) |
| Hotta | 1.45 (1.23 to 1.72) | 1.52 (1.03 to 2.24) | 1.44 (1.22 to 1.69) |
| Li | NA ^d^ | NA | NA |
| Peeters | 1.34 (1.01 to 1.78) | 1.47 (1.02 to 2.11) | 1.34 (1.07 to 1.69) |
| Jacobsson_2008 | 1.23 (0.94 to 1.62) | 1.49 (1.09 to 2.03) | 1.29 (1.04 to 1.60) |
| Muler | 1.24 (0.86 to 1.80) | 1.47 (0.97 to 2.23) | 1.30 (0.98 to 1.73) |
| Andreasen_a | 1.38 (1.18 to 1.61) | 1.58 (1.31 to 1.90) | 1.40 (1.23 to 1.58) |
| Andreasen_b | 1.23 (1.08 to 1.39) | 1.37 (1.17 to 1.61) | 1.24 (1.12 to 1.38) |
| Andreasen_c | 1.27 (0.78 to 2.09) | 1.52 (0.83 to 2.80) | 1.32 (0.88 to 1.96) |
| Price | 1.63 (1.26 to 2.11) | 2.80 (1.99 to 3.92) | 1.83 (1.48 to 2.26) |
| Legry | 1.25 (1.03 to 1.52) | 1.29 (1.03 to 1.62) | 1.24 (1.06 to 1.44) |
| Gonzalez | 1.51 (1.07 to 2.13) | 1.55 (1.02 to 2.38) | 1.47 (1.11 to 1.94) |
| Zabena | 2.25 (1.24 to 4.10) | 3.14 (1.60 to 6.17) | 2.32 (1.43 to 3.74) |
| Tabara | 1.62 (1.21 to 2.18) | 1.96 (0.92 to 4.15) | 1.61 (1.21 to 2.14) |
| Willer | NA | NA | NA |
| Jacobsson_2009 | 1.50 (0.83 to 2.73) | 1.07 (0.51 to 2.23) | 1.31 (0.83 to 2.05) |
| Karasawa | 1.25 (1.05 to 1.48) | 1.43 (0.98 to 2.08) | 1.25 (1.06 to 1.47) |
| Liu | 1.39 (1.02 to 1.89) | 2.04 (0.66 to 6.28) | 1.39 (1.02 to 1.88) |
|  |  |  |  |
| Pooled OR | **1.36 (1.29 to 1.42)** | **1.54 (1.41 to 1.69)** | **1.36 (1.30 to 1.41)** |
|  | | | |
| **rs1421085** | | | |
| Dina_a | 1.80 (1.51 to 2.15) | 1.81 (1.52 to 2.17) | 1.70 (1.49 to 1.94) |
| Dina_b | 1.50 (1.20 to 1.86) | 1.74 (1.38 to 2.20) | 1.52 (1.29 to 1.80) |
| Dina_c | 1.72 (1.31 to 2.26) | 1.65 (1.22 to 2.22 ) | 1.60 (1.30 to 1.98) |
| Dina_d | 1.41 (1.07 to 1.86) | 1.34 (1.01 to 1.77) | 1.33 (1.08 to 1.64) |
| Dina_e | 1.94 (1.40 to 2.67) | 2.07 (1.49 to 2.88) | 1.86 (1.46 to 2.37) |
| Peeters | 1.42 (1.07 to 1.88) | 1.46 (1.03 to 2.06) | 1.38 (1.10 to 1.74) |
| Attaoua | 2.27 (1.28 to 4.03) | 2.08 (1.15 to 3.77) | 2.01 (1.31 to 3.10) |
| Meyre_FC | 1.46 (1.12 to 1.90) | 1.42 (1.05 to 1.90) | 1.39 (1.13 to 1.71) |
| Meyre_FA | 1.59 (1.03 to 2.44) | 1.59 (1.03 to 2.46) | 1.52 (1.10 to 2.09) |
| Meyre_SA | 1.19 (0.91 to 1.56) | 1.18 (0.88 to 1.58) | 1.17 (0.95 to 1.44) |
| Meyre_GC | 1.82 (1.35 to 2.45) | 1.57 (1.19 to 2.09) | 1.60 (1.30 to 1.98) |
|  |  |  |  |
| Pooled OR | **1.59 (1.45 to 1.74)** | **1.60 (1.45 to 1.76)** | **1.52 (1.39 to 1.65)** |
|  | | | |
| **rs8050136** | | | |
| Hinney | 1.76 (1.33 to 2.33) | 1.97 (1.41 to 2.74) | 1.73 (1.39 to 2.16) |
| Song_AP | 1.03 (0.57 to 1.83) | 1.55 (0.48 to 5.04) | 1.05 (0.61 to 1.83) |
| Song_B | 0.91 (0.70 to 1.18) | 1.08 (0.78 to 1.48) | 0.97 (0.78 to 1.20) |
| Li | NA | NA | NA |
| Grant_C | NA | NA | NA |
| Grant_AA | NA | NA | NA |
| Thorleifsson | NA | NA | NA |
| Liu | 1.47 (1.08 to 2.00) | 1.63 (0.49 to 5.46) | 1.47 (1.08 to 1.99) |
| Cheung | 1.49 (1.15 to 1.95) | 2.35 (0.91 to 6.11) | 1.50 (1.15 to 1.95) |
|  |  |  |  |
| Pooled OR | **1.32 (1.01 to 1.71)** | **1.56 (1.07 to 2.26)** | **1.34 (1.04 to 1.71)** |
|  | | | |
| **rs17817449** | | | |
| Dina_a | 1.71 (1.44 to 2.04) | 1.88 (1.57 to 2.25) | 1.68 (1.47 to 1.92) |
| Dina_b | 1.65 (1.33 to 2.06) | 2.13 (1.69 to 2.70) | 1.74 (1.47 to 2.05) |
| Dina_c | 1.81 (1.38 to 2.38) | 1.71 (1.26 to 2.31) | 1.67 (1.35 to 2.06) |
| Dina_d | 1.27 (0.97 to 1.66) | 1.33 (1.00 to 1.78) | 1.26 (1.03 to 1.55) |
| Dina_e | 1.93 (1.39 to 2.68) | 1.95 (1.41 to 2.69) | 1.82 (1.43 to 2.31) |
| Price | 1.70 (1.31 to 2.21) | 2.76 (1.97 to 3.85) | 1.88 (1.52 to 2.32) |
|  |  |  |  |
| Pooled OR | **1.66 (1.50 to 1.83)** | **1.90 (1.61 to 2.26)** | **1.66 (1.50 to 1.84)** |
|  | | | |
| **rs1121980** | | | |
| Hinney | 1.93 (1.44 to 2.59) | 1.98 (1.43 to 2.73) | 1.83 (1.46 to 2.29) |
| Hotta | 1.41 (1.19 to 1.66) | 1.40 (0.99 to 1.99) | 1.39 (1.19 to 1.62) |
| Renstrom | NA | NA | NA |
|  |  |  |  |
| Pooled OR | **1.61 (1.19 to 2.18)** | **1.68 (1.20 to 2.36)** | **1.57 (1.20 to 2.06)** |

^a^: Dominant genetic model;

^b^: Recessive genetic model;

^c^: Generalized OR [1];

^d^: Not available.

1. Zintzaras E: **The Generalized Odds Ratio as a Measure of Genetic Risk Effect in the Analysis and Meta-Analysis of Association Studies**. *Stat Appl Genet Mol Biol* 2010, **9**.

**Figure S1.** Meta-regression of mean control BMI and genetic effect using an allelic comparison model in rs9939609. There was a significant correlation between mean control BMI and genetic effect (*P* = 0.007).

**Figure S2.** Forest plot of the association between rs9939609 and obesity in additive model


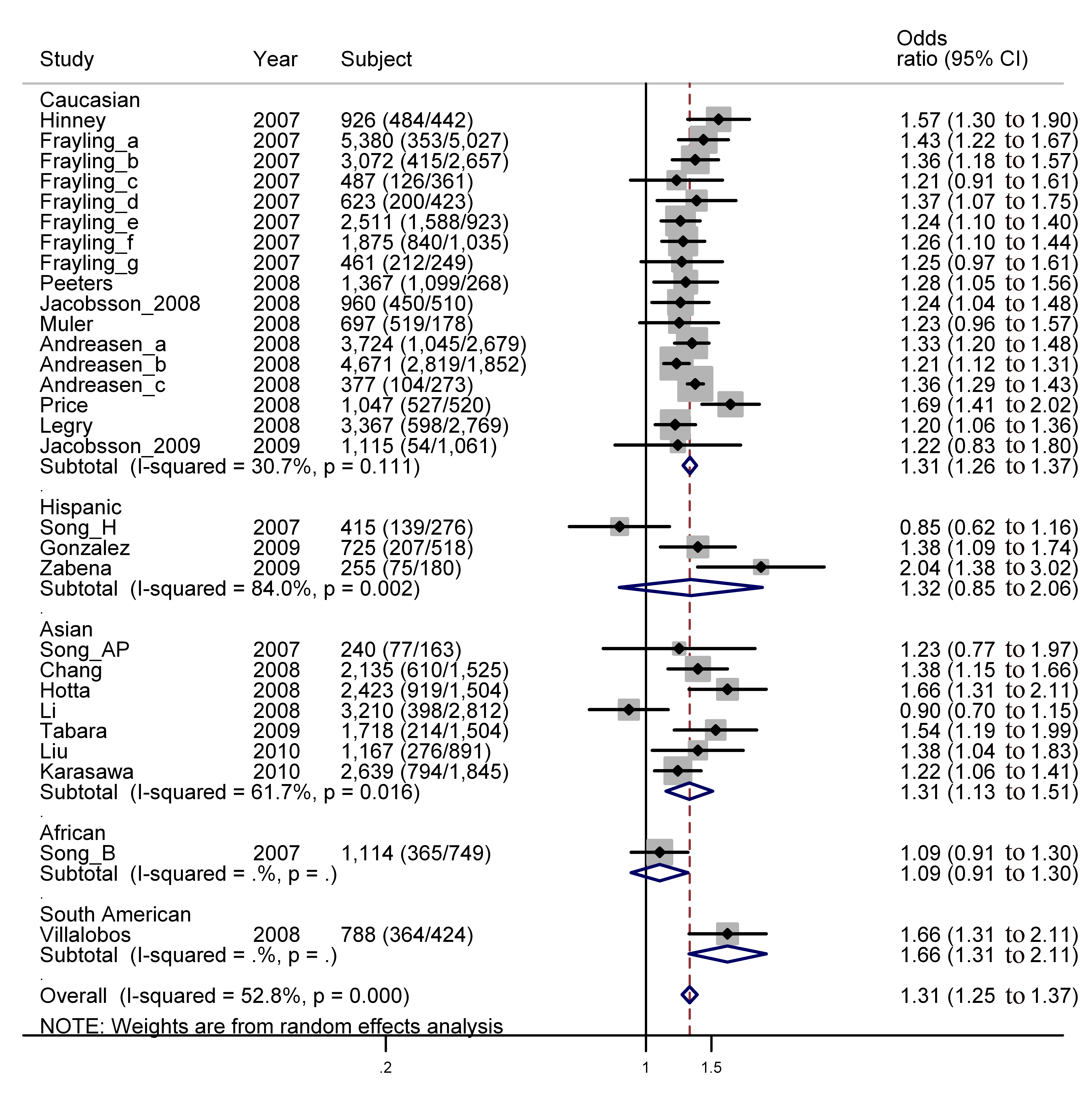


**Figure S3.** A comparison of the effect size across various ethnic populations between different SNPs and obesity risk


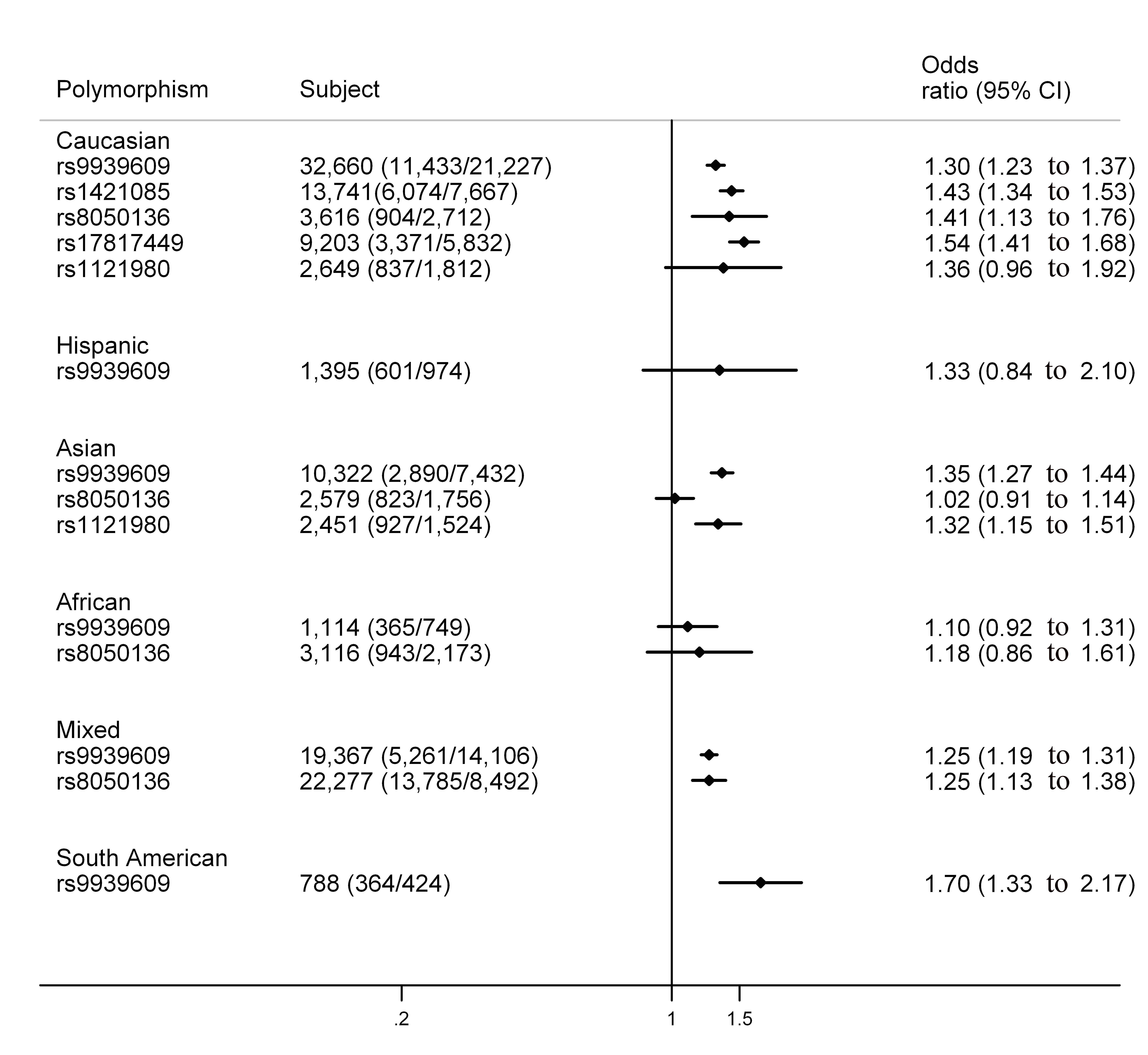


**Figure S4.** Forest plot representing the association between rs9939609 and obesity risk under per-allele comparison after exclusion of the three children studies


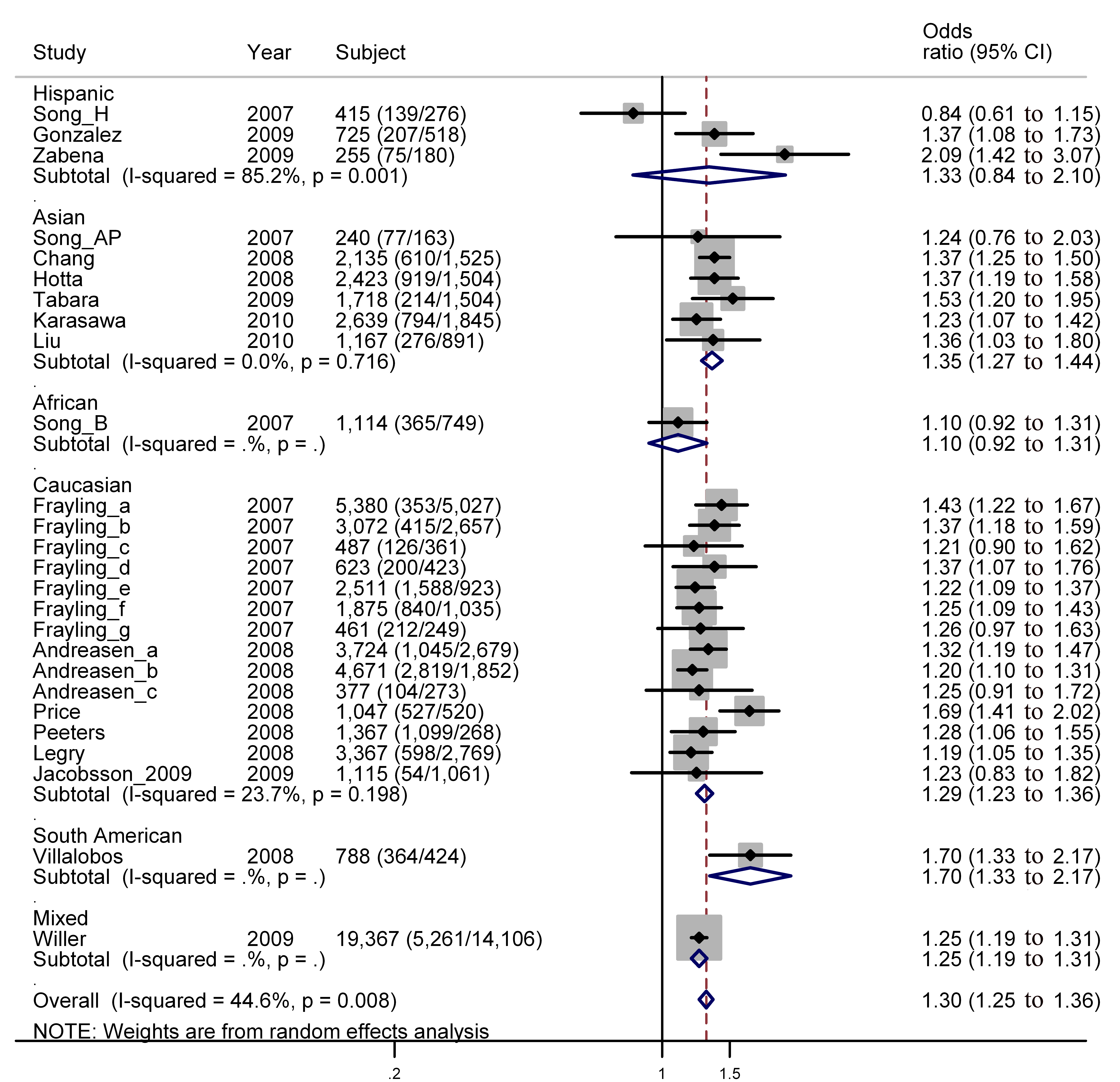

Supplement: Additional file 1 — Table S1, Figure S1, Figure S2, Figure S3, and Figure S4. [file 1741-7015-9-71-S1.DOC]
